# Trends in maternal and neonatal mortality in South Africa: a systematic review protocol

**DOI:** 10.1186/s13643-017-0560-1

**Published:** 2017-08-17

**Authors:** Damian J. Damian, Bernard Njau, Ester Lisasi, Sia E. Msuya, Andrew Boulle

**Affiliations:** 1 School of Public Health and Family Medicine, University of Cape Town, Cape Town, South Africa; 2 Institute of Public Health, Kilimanjaro Christian Medical University College, Moshi, Tanzania

**Keywords:** Maternal mortality, Neonatal mortality, Millennium development goals, South Africa

## Abstract

**Background:**

Measuring and monitoring progress towards Millennium Development Goals (MDG) 4 and 5 requires valid and reliable estimates of maternal and neonatal mortality. In South Africa, there are conflicting reports on the estimates of maternal and neonatal mortality, derived from both direct and indirect estimation techniques. This study aims to systematically review the estimates made of maternal and neonatal mortality in the period from 1990 to 2015 in South Africa and determine trends over this period.

**Methods:**

For the purpose of this review, searches for eligible studies will be conducted in MEDLINE, Africa-Wide Information, African Index Medicus, African Journals Online, Scopus, Web of Science and CINAHL databases. Searches will be restricted to articles written in English and presenting data covering the period between 1990 and 2015. Reference lists of retrieved articles will also be screened for additional publications. Three independent reviewers will be involved in the study selection, data extractions and achieving consensus. Study quality and risk of bias will thereafter be assessed by two authors. The results will be presented as rates/ratio with their corresponding 95% confidence/uncertainty intervals.

**Discussion:**

Identifying trends in maternal and neonatal mortality will help to track progress in MDGs 4 and 5 and will serve in evaluating interventions focusing on reducing maternal and child mortality in the country. This study will, in particular, provide the context for understanding inconsistencies in reported estimates of maternal and neonatal mortality by considering estimation methods, data sources and definitions used.

**Systematic review registration:**

PROSPERO CRD42016042769

## Background

Monitoring progress towards Millennium Development Goals (MDGs) 4 and 5 (reducing child and maternal mortality, respectively, between 1990 and 2015) requires valid and reliable estimates of maternal and child mortality in the country. Various methods for measuring and estimating maternal and neonatal mortality have been developed, tested and widely used [1–8]. Estimating these outcomes in developing countries is challenging due to the lack of accurate, valid and reliable data [9–14].

Recent estimates from the United Nations Inter-agency Group for Child Mortality Estimation (UN-IGME) and Maternal Mortality Estimation Inter-agency Group (MMEIG) indicated that South Africa did not achieve the MDG 4 and 5 targets by 2015 [15, 16]. Considering other African countries which did not meet MDG 4 and 5 targets, only South Africa had conflicting estimates of maternal and neonatal mortality reported by different sources [15–20].

South Africa is unusual among developing countries in that national facility-based mortality audits are carried out for maternal, perinatal and child deaths [21, 22]. Estimation of maternal and neonatal mortality in the country is often based on the National Confidential Enquiry into Maternal Deaths (NCEMD) which records maternal deaths and the Perinatal Problem Identification Program (PPIP) which records stillbirths and neonatal deaths [22–24].

The country provides unique opportunities to estimate these outcomes empirically, analytically or through modeling, by having multiple data sources with wide coverage [1–5, 23, 25, 26]. However, there are widely divergent estimates, wherein the two most frequently cited estimates are from institutional reporting and WHO metrics, which makes it difficult to both understand trends in these outcomes and assess the successes or failures of interventions focusing on reducing maternal and child mortality in the country over the past decade. The reasons for divergent estimates between institutional reporting and WHO metrics, or among global metrics, are partly explained by estimation approaches and quality of data. There have been limited attempts to review maternal and neonatal mortality estimates in South Africa to facilitate understanding of trends during the MDG period.

This review is aimed at providing an overview of estimates of maternal and neonatal mortality for the period 1990 to 2015 in South Africa and determining the temporal trends during this period. Moreover, it aims to provide the context for understanding inconsistencies in reported estimates of maternal and neonatal mortality by the institutional reporting and the global metrics by considering estimation methods, data sources and definitions used.

## Methods

This will be a systematic review of trends in maternal and neonatal mortality estimates for the period 1990 to 2015 in South Africa.

### Eligibility criteria

The population for eligible studies will include pregnant women and neonates (a human infant from the time of birth to the 28th day of life) for ascertaining maternal and neonatal mortality respectively. All studies which are nationally representative, reports providing national-level data (and trends thereof) and vital registration data will be eligible for this review. Searches will be restricted to studies being conducted in South Africa or which have used South African data, and multicentre studies including South Africa, reporting data covering the period 1990 to 2015. No restrictions on the date of publication will be made.

### Information sources

Separate searches for the two outcomes (maternal and neonatal mortality) will be conducted in the following electronic databases: MEDLINE, Africa-Wide Information, African Index Medicus, African Journals Online, Scopus, Web of Science and CINAHL. No restrictions on the date of publication will be made. Additional searches for conference abstracts and proceedings will be conducted in this review. Reference lists of retrieved articles will also be screened for additional publications. Contacts with experts in the field of study will be made to identify additional relevant articles.

### Search strategy

Keywords to be used for the publication search include the following: “maternal mortality”, “maternal death”, “neonatal mortality”, “neonatal death”, “estimate”, “rate”, “ratio” and “South Africa”, by using the Boolean operators “OR” or “AND” (see the Appendix). Searches will be restricted to articles written in English and reporting data covering the period from 1990 to 2015. Table 1 provides﻿ initial search strategy for medline.

### Study records

#### Data management

Search outputs will be managed in Mendeley reference manager. Any duplicate records will be removed before the selection process takes place. When the same article has been captured in different journals, or the same results have been presented with different main authors, the most detailed publication will be selected for review.

#### Selection process

Three independent reviewers will be involved in the screening and selection of articles. This will involve an assessment of articles based on titles and abstracts using Covidence software (https://www.covidence.org/). In the case of insufficient information in the title and abstract, the full text of the specific article will be retrieved and assessed. For an article to be eligible for inclusion in the systematic review, two reviewers must agree to include it. A third reviewer will be consulted in case of any difference of opinion between the two reviewers. This will follow when they fail to reach a consensus after joint examination of the different views.

#### Data collection process

Analysis of the full text will be conducted for all eligible articles. Two authors will extract data independently using a pre-agreed data abstraction template. In the case of discrepancies between authors, consensus will be sought before involving a third author for tie-breaking. During the data extraction process, authors/investigators will be contacted if there is insufficient information/data provided in the article.

### Data items

For eligible studies, the following information will be extracted: first author’s name, year of publication, year of death (maternal and neonatal), number of pregnant women, number of live births, maternal deaths, neonatal deaths, definition of maternal death, definition of neonatal death, maternal mortality ratio/rate (if reported, and by year), neonatal mortality rate (if reported, and by year) and an indicator variable whether the records are complete.

### Outcomes and prioritization items

The main outcome in this review includes maternal and neonatal mortality. Maternal death/mortality will be defined as the death of a woman while pregnant or within 42 days of termination of pregnancy, irrespective of the duration and site of the pregnancy, from any cause related to or aggravated by the pregnancy or its management but not from accidental or incidental causes (ICD-10). Maternal mortality ratio (MMR) will be defined as the number of maternal deaths per 100,000 live births (ICD-10). Neonatal death/mortality will also be defined as the death of live-born within the first 28 days of life. Neonatal mortality rate will be defined as the number of infant deaths within the first 28 days of life per 1000 live births.

### Risk of bias in individual studies

Two authors will assess the study quality based on the following quality assessment criteria: (1) definition of maternal mortality, (2) definition of neonatal mortality, (3) completeness of ascertainment of maternal and neonatal mortality, (4) completeness of ascertainment of live births, (5) sampling technique and (6) data quality. Studies will be assessed based on each criterion and will be rated as “high risk of bias” or “low risk of bias” accordingly. Studies with a rating of “high risk of bias” in any criterion will be assigned an overall rating of “high risk of bias” while overall rating of “low risk of bias” will only be assigned in studies with “low risk of bias” in all criteria.

### Data synthesis

Data will be presented by year as rates/ratio for both maternal and neonatal mortalities with their corresponding 95% confidence/uncertainty intervals. Furthermore, the findings will be presented graphically using a scatter plot to depict trends, and comparisons will also be described according to the estimation techniques used. Accounting for quality and similarity of eligible studies, meta-analysis will be carried out to determine the pooled estimates of maternal and neonatal mortality respectively. Sensitivity analysis will be conducted to investigate the robustness of the findings in relation to risk of bias and the analytical approaches employed (random effect vs. fixed effect meta-analysis)

### Publication bias

Risk of publication bias will be assessed using a funnel plot for each outcome (maternal and neonatal mortality) separately. Furthermore, an extensive search strategy will be employed to identify additional relevant publications in grey literature in order to minimize the risk of publication bias.

## Presenting and reporting of results

The presentation and reporting of results in this review will follow the systematic review reporting standard (PRISMA-P) [27]. To ensure transparency, a PRISMA flow chart will be used and a table indicating all included studies will be presented in the report [28]. The reasons for study exclusions will be explained clearly and documented. In case it is not possible to obtain a pooled estimate, findings from individual studies will be narrated and described.

## Discussion

Tracking progress in MDGs 4 and 5, and evaluating interventions focusing on reducing maternal and child mortality in the country, requires reliable and accurate estimates of maternal and neonatal mortality. In South Africa, there are divergent estimates of maternal and neonatal mortality obtained from institutional reporting and global metrics. More accurate estimates of neonatal and maternal mortality are expected to be derived from pooled estimates from studies which are nationally representative, reports providing national-level data (and trends thereof) and vital registration data with high quality and low risk of bias. This review will give overviews of maternal and neonatal mortality estimates over the MDG period (1990–2015) and time trends. This study will also provide context for understanding the divergence in different maternal and neonatal mortality estimates in the country.

## Appendix

**Table 1:** Literature review search strategy

Database	Search terms	Retrieved	Screened	Used
PubMed	((“mothers”[MeSH Terms] OR “mothers”[All Fields] OR “maternal”[All Fields]) OR (“infant, newborn”[MeSH Terms] OR (“infant”[All Fields] AND “newborn”[All Fields]) OR “newborn infant”[All Fields] OR “neonatal”[All Fields])) AND ((“mortality”[Subheading] OR “mortality”[All Fields] OR “mortality”[MeSH Terms]) OR (“death”[MeSH Terms] OR “death”[All Fields])) AND (estimation[All Fields] OR estimates[All Fields]) AND (“South Africa”[Mesh] OR (“south africa”[MeSH Terms] OR (“south”[All Fields] AND “africa”[All Fields]) OR “south africa”[All Fields])) AND ((“1990/01/01”[PDAT] : “3000/12/31”[PDAT]) AND “humans”[MeSH Terms] AND English[lang])			

## Abbreviations

MDG: Millennium Development Goal
MMR: Maternal mortality ratio
PRISMA-P: Preferred Reporting Items for Systematic review and Meta-Analysis Protocols
